# Abbreviated Report on Obstetrics

**Published:** 1867-07

**Authors:** Hiram Nance

**Affiliations:** Kewanee, Ill.


ARTICLE XXIX.

### ABBREVIATED REPORT ON OBSTETRICS.

By HIRAM NANCE, M.D., Kewanee, Ill.

Read before the M. T. Medical Society, Monmouth, Ill.

In writing a report on the Practice of Midwifery, to be read to any medical association whose meetings last only one day, and whose members come from five populous counties, I would be doing injustice to its members in making it lengthy. Short and sweet should be the motto on such ocasions, and this shall characterize this essay.

We are all familiar with the divisions of midwifery, or its more immediate conger, *labor:* into natural, or unnatural, or preternatural, rapid labor, tedious labor, instrumental labor, etc., etc. In writing this report, I may give *my practice and experience* in this branch of our profession, taking a retrospect, or a review, of my own personal past experience.

The practice of midwifery is certainly reduced to a science amongst the more elevated portions of society—pity I could not say the same in regard to all classes. What I mean by this is, that, usually, the more intellectual and intelligent avail themselves of the services of physicians during the stage of parturition. And amongst this class, we find that the mortality originating from malpositions and puerperal diseases, of various kinds, is much less than among the less intellectual and poor. Does not this prove conclusively that the office of the physician is not a sinecure, or that his valuable services could not be dispensed with with impunity? It is true, that in many, if not in most cases of labor, our services could be dispensed with, and that any ordinary nurse or matronly lady could make gentle pressure over the uterine tumor during the immediate transit of the child from the mother into the world, thus preventing hemorrhage by the immediate contraction of the womb. Almost any nurse ought, also, to be able and wise enough to examine for the umbilical cord, and ascertain if it is around the child's neck, or is being compressed in any way that would endanger the life of the infant; she should also know enough to make the necessary efforts to resuscitate the child in case it is born asphyxiated, such as dashing cold water on it, HALL'S *ready method*, performing the act of artificial breathing, etc., etc. These troubles occur in the simplest cases of labor, and if our nurse is destitute of this small amount of obstetrical knowledge, she is not a fit person to occupy the lying-in room without a superior, in the person of a physician, being placed over her, in every case of obstetrics. As I remarked before, this branch of our profession is a science, and none but scientific persons should take the weighty responsibility upon themselves of engaging in its practice. One hour, fifteen minutes, yes, one minute, is sometimes enough to apply our skill and save a valuable life or lives.

The obstetrician should be a wise man; one well and thoroughly informed in his profession. He should know when to act and how to act, in all simple as well as difficult cases. In taking charge of an obstetrical case, the physician should, at a very *early stage* of labor, insist upon a digital examination. This, when accomplished, will not only allay your anxiety, but that of your patient, when you inform her that *all is right.* If the presentation is found to be natural, then you have but little to do but cheer your patient up, until the last stage of labor; but if you find a malpresentation, then you hold yourself ready to act when the proper time arrives—remain in the room or adjoining one, subject to call at any moment, and make frequent but not officious examinations, to know when the proper time arrives for your learned skill to be employed. Indifference and timidity, connected with ignorance, has caused the death of many a fond wife and loving mother. You are not a fit man to practice obstetrics, if you are not patient yet energetic, and wise enough to know the exact moment when to lend the necessary aid to assist your patient out of a trouble, the most painful and serious that befalls our gentler sex.

By labor, we mean the delivery or the expulsion of the fœtus and its appendages. Labor is the consequence of conception; and the time occupied in utero-gestation is nine calendar months, or from 270 to 274 days. When interrogated by a lady, to know at what time her *accouchement* will take place, I enquire when she had her last menstrual flow, and from this time add one or two days more, and, usually, in nine months from that time she will fall in labor. Some obstetricians count two weeks from the last menses, and then add nine months; but I think our prognosis will be much more likely to be filled if we calculate as I first mentioned.

Labor is divided, with some degree of propriety, into four stages:—The first stage is known by a mucous or sanguineous discharge taking place from the vagina, from one to two or three days before true labor pains commence, also, we find at the same time, wandering pains about the back and loins, sometimes extending down the thigh. The second stage is known by the pains increasing in frequency and efficiency, commencing in the bowels and lumbar region and extending to the region of the womb, the dilatation of the os uteri, the protrusion of the bag of waters in the vagina, and the rupture of the same. When the liquor amnii has escaped without the interference of any external aid, and the presenting part of the child is in or or about entering the vagina, the second stage of labor is completed. Then comes the third and important part, or the delivery of the fœtus, known by the firm contractions of the abdominal muscles and the uterus, the head engages in the pelvic cavity, the occiput generally being situate above the arch of the pubis or the left acetabulum, and the face opposite the sacrum of the mother. The head now, after some severe pains, presents itself at the vulva, powerfully distending the perineum; in a short time the head clears its confined state and is in the world; a minute or two elapses, and one or two severe pains accomplishes the delivery of the child. It now remains to separate the child from the mother, which being done, in ten or fifteen minutes slight pains return. By making the necessary pressure on the uterine tumor and gentle traction on the cord, we have the pleasure of having delivered our patient of the secundines, which include not only the placenta, but the different membranes which envelope the fœtus in utero; this delivery I would class as the fourth stage of labor.

Some obstetricians class the last throes of labor, with the delivery of the placenta, as the fourth stage; but I prefer to call the fourth stage the removal of the appendages, independent of the removal of the fœtus, for the reason that the delivery is not completed until this is over; and we are not justified in leaving the bedside of our patient until this is entirely completed, and we have ascertained, by examination, that the womb is firmly contracted. Immediately after, or just at the time the babe is being born, the hand should be applied over the hypogastric region, to ascertain if the womb is contracted; if it is not, no effort should be made to deliver the placenta, for if we do attempt it, a fatal hemorrhage might ensue.

I was called in consultation, a few years ago, to see a patient, who, the messenger said was dying; when I arrived, I found it too true, it was really the case. I interrogated the attending physician: he said, that morning he was called to see her, found her in labor, (the labor was premature, being about the sixth month,) that she lingered from about 2 or 3 o'clock A.M. until the afternoon of the same day, that the afterbirth did not come away readily, and that he found it necessary to make slight traction on the cord and introduce two or three fingers to remove it; that, in doing so, he accomplished it and left the room, and was in a very short time summoned to his patient, to find her very weak, pale, and faint. He immediately ordered carb. ammo., opii, brandy, etc., but finding reaction did not come on, he became alarmed and dispatched a messenger for me. When I arrived, she was really dying; pulse small and thready; countenance sunken and exsanguined; slow and difficult breathing; very uneasy, rolling from side to side; yet the intellect was perfect. On applying my hand over the bowels, I found them as large, if not larger than before the *accouchement*—the story, to my mind, was immediately told, *viz.*:—concealed, or internal, uterine hemorrhage. My advice to you is, never leave a patient until the uterus is firmly contracted.

If you are an obstetrician, you practice for the good of your patient, and your own reputation; you should not leave your patient, even if everything has been favorable, in less time than from 30 to 60 minutes. Had the attending physician in this case remained by the side of his patient, the consequences might have been different. The first symptom of the loss of blood, in nine cases in ten, is a desire for a drink of water; then, paleness, sickness, and fainting. These symptoms should rouse the fears of any physician, whether hemorrhage appears externally or not.

What course of treatment will you adopt if, upon examination, you find there is no hemorrhage externally? If there is no hemorrhage externally, and the symptoms that I have described are existing, your patient is *flowing*. How are you to know this? Apply your hand over the hypogastric region, and you will find that the uterus is not contracting, but that it may be nearly, if not quite, as large as it was before the birth of the child.

If you now, under these urgent symptoms, pursue a *dilly-dally* course of treatment, your patient in a very short time will be in extremity, and you will reflect upon yourself, and feel very much chagrined that your treatment was not more active and directly addressed to her wants. In brief, I would urge that no time is to be lost; administer a portion of ergot, sit down by your patient, apply one hand over the hypogastric region and make firm pressure, in order to assist the uterus to contract. If, under this treatment, no contraction takes place, introduce the other hand in the uterus and press the knuckles or back part of the hand against the orifices of the bleeding vessels, at the same time keep making pressure on the outside; continue this treatment, and in a short time you will usually find the uterine tumor becoming harder and harder and the coagula coming away, and then the hemorrhage will cease. If this treatment does not succeed, you should also use cold water or water and vinegar injections in the womb, or pour iced water over the hypogastric region. The internal treatment should consist of tinct. opii, ergot, carb. ammo., brandy, etc., according to the best judgement of the practitioner. The treatment that I have described apply to cases where the placenta has been delivered. If the placenta is undelivered and retained, as in irregular or hour-glass contractions, of course it should be removed.

In the summer of 1866, a man, residing six miles west, came to consult me in regard to his wife. He called upon me about 8½ o'clock A.M. He said, about 5 o'clock that morning, his wife had been confined and delivered of a nice pair of twins, but the afterbirth did not come away; he wanted me to give him something to bring it away. He said a midwife was with her, and he thought that the case was not urgent, and she thought I could send something that would bring it away. I frankly told my friend of *Erin* that I was fearful the case was a serious one, and that I had better go out immediately; by so doing, I might save his wife much suffering, and, probably, her life. He *squirmed* and hesitated, and finally said, "they" told him to get some medicine. I informed him that I could send some medicine if he desired it, but that I was of the opinion that medicine would be of no avail, but that she would require the assistance of some physician to remove the placenta, if it had been retained four or five hours. After stating these things to him, the poor man was *penurious* and simple enough to leave me and go home without either taking medicine or engaging my services, and letting his wife lie in torment until about 2 o'clock P.M. of the same day. About this hour, a messenger came, in a great hurry, for me to go immediately and see Mrs. L., as they feared she would not live. When I arrived, I found the old midwife there. She was wise enough not to do any harm, but sat by the patient, consoling her that "all would soon be right, as the doctor had come."

On examining the patient, I found her with a flushed face, full and rapid pulse, and a tendency to delirium; on applying the hand over the bowels, I found the uterine tumor hard and irregular; both umbilical cords were hanging out of the vagina, and, on digital examination, I could not find the placenta; I diagnosed irregular or hour-glass contraction of the womb, and the placenta in the upper chamber. I arranged the patient in a suitable position for operation for removal, administered chloroform, introduced my hand, found things as I had anticipated, grasped hold of the placenta, and, with the other hand applied externally over the placenta, gradually brought it away. It proved to be a large double placenta, firmly attached to each other. It being, delivered, I administered an opiate to my patient. When about leaving, I told them I would call the next day and see her, as I feared she would not get along well. He replied that, if any trouble occurred he would let me know; then grudgingly paid me my fee, after asking me to reduce it. Let me advise you, in like cases, to charge well for your services, and they will be better appreciated than when a mere nominal sum is demanded.

During the last six years and nine months that I have resided in Kewanee, only two cases of arm presentation have come under my immediate notice, and both of them were first under the care of midwives, secondly, under the care of other physicians, and, lastly, under my own care. The first case was a lady, about 40 years of age, with her sixth child. She was taken ill in the night and the midwife sent for, who encouraged her that she would soon get through. She continued giving this encouragement until about 3 o'clock of the next day, when, the lady not getting any better, the parties became restless and uneasy, and, having lost confidence in the midwife, proposed sending for me, and did so, but I was not at home. A homœopathist was then obtained; he went, and found the arm in the vagina, with the hand out, the patient having very hard pains, forcing the arm out as far as the pelvis would permit. He manipulated with it in this condition for several hours. It seems that he had no correct idea how to deliver the poor woman; I think he must have pulled on the arm, hoping to pull it out in that way. After exhausting his *strength* and skill, all parties, and himself in particular, became satisfied that labor could not be accomplished without more skill than he possessed. He (the homœopathist) advised them to go for me as soon as possible, and tell me to bring my instruments. I obeyed the summons, not as a consulting physician, but because I had first been sent for. When I arrived, I asked the "doctor" what the difficulty was. He said, an "arm presentation." I then said to him, "what do you want with instruments?" "Oh!" said he, "to amputate the arm." I mildly told him he had no authority for such interference, and explained, in brief, that turning was the only treatment in such cases, where the pelvis was ordinarily capacious.

I then examined, and found the mother almost exhausted; constant pain; pulse 120; tenderness over the uterine region; and other symptoms of inflammation. On examination of the fœtus, I found the arm out, and it perfectly black. I administered chloroform, and turned and delivered her in fifteen or twenty minutes. The poor woman seemed to rally some after it was through, but the pulse still remained quick and the bowels tender. The mischief had been done; the long-continued pressure of the child in the pelvis and the arm in the vagina had set up an inflammation which spread to the peritoneum, and in six or seven days she died with puerperal fever, or metritis, connected with peritonitis. Had I been called at a proper time, I have no doubt but that I could have saved the life of this poor woman. It is humiliating to think that people will persist in employing such men, who do not understand even the first principles of midwifery, and thus sacrifice many valuable lives.

The second case of arm presentation was that of an Irish-woman, aged 33, with her fifth child. She was taken sick in the night and sent for an Irish midwife, who came and continued with her until 3 o'clock P.M. of the next day. She then concluded that the woman could not get through without the aid of a physician. Dr. SCOTT, of this place, was called and found the arm entirely out and the patient in great pain, almost continually. As the pains were so severe and almost without remission, he saw it was useless to attempt turning without the assistance of some other physician, and he requested that I be sent for, which was readily agreed to. The doctor administered chloroform, and I proceeded to turn, which I did with but little trouble in ten or fifteen minutes time. The child was dead; the mother got along without an untoward symptom.

I have not been fortunate enough in my practice to meet with an arm presentation at the proper time to turn, but have always found them in the situation of the two cases just related, *viz.:*—the arm forced down and the axilla resting on the brim of the pelvis; the waters all discharged; and the woman in almost continued pain. Could I select the proper time to turn, in arm presentations, I would wait until the os uteri was sufficiently dilated, then introduce the hand corresponding with the position of the feet in utero, rupture the membranes, grasp the feet, or, if I could not reach both, bring down one, and deliver as I would an ordinary footling case.

Feet and breech presentations occur so often, that it is hardly required that I should call the attention of the Society to them. They occur about every thirtieth labor. I have the satisfaction of stating to you that I never lost an infant in my practice that presented either head or breech. Some authors state that the mortality in these cases will average one-third of them. I manage them by letting them alone until the feet and breech are born, then ascertain if the cord is being compressed, if the pulsation is weak and feeble, I bring down a coil of it to prevent compression, then make traction on the infant by folding a towel around it and using gentle force; if the face is not coming down opposite the sacrum and perineum, I turn the child so it will, then, as soon as I can, I introduce my finger in the mouth and bring it into the world as soon as consistently practicable. I failed to mention that the arms should be brought down over the face, and in doing this great care should be used or we may fracture the humerus. An accident of this kind once occurred to me. RAMSBOTHAM quotes several cases of this kind, and cautions us in regard to it, but, notwithstanding his caution, it occurred to me. I immediately dressed the little arm, and in ten days it was as well as ever.

Great excitement was caused in our village, a year or two ago, by a physician having care of a lady, who was so unfortunate as to have a footling case. It was stated that he let it hang by the neck, undelivered, half an hour, and in this position had to have counsel to help him out of the difficulty. Difficulties may occur with any of us; but is an obstetrician justified in such a case not to do better than this, where the lady is well formed and the child of ordinary size?

I reiterate that no man nor woman should engage in the practice of midwifery until he or she has just and clear views of the process of parturition, and is freely prepared to act in all cases, either simple or complicated. And I believe it to be our bounden duty to expose all men or women who are not competent to take charge of such cases. If we attempt to practice a profession and are not well informed in it, we are responsible to our fellow-men and to our God—and shall we by our taciturnity become the accomplices, aiders, and abettors of their ignorance?

I have seen but few cases of face presentation in my own practice, or in the practice of others. Eight or ten years ago, I met with a case, in consultation with Dr. CLARK, of Galva. Our only treatment was *nature* and *time*, and the woman got along well, and, so far as I remember, had a living child.

On the 29th Sept., last, I was sent for to see Mrs. B., residing four miles from this place. I arrived about 9 o'clock A.M. She had been sick since 12 or 1 o'clock. On examination, I diagnosed face presentation, with the forehead presenting at the symphysis pubis, and the chin at the sacrum. The os was fully open, but no advancement was made, yet the woman had frequent and severe pains. It was the seventh labor, and she had always, but once, been confined and delivered without the assistance of a physician, and this time she had attempted the same, but the woman in attendance readily yielded the case when she came to the conclusion that the presentation was *preternatural.* The uterus was so firmly contracted I could not make any change in its situation, and, therefore, was obliged to apply the forceps, which I did with great difficulty, but finally succeeded, and delivered her of a very large dead child. Had the child been reversed, with the forehead resting on the perineum and the chin at the pubis, I think it could have been born without instrumental interference, but as it was, it was an impossibility.

Face presentations, as a general rule, should be left to nature, they only require, in the language of Dr. GOOCH, "more time, more labor pains, and more patience than a natural presentation."

By transverse or cross births, we mean when any other part presents except the head, feet, or breech. A peculiar presentation occurred to me during the month of April, of which I herewith hand you an ambrotype, taken from a plate in RAMSBOTAAM'S Process of Parturition, (plate No. 127.) The process of ambrotyping or photographing, as you all know, reverses the order of the picture, and instead of finding the vertex in the cavity of the left ilium it was in the right; instead of the foot being down in the vagina, as here represented, the toes, up to the instep, had fixed themselves over the arch of the pubis, while the heel alone presented in the vagina; while, at the same time, the vertex occupied the iliac fossa. The hand was presenting as you here see, and the umbilical cord likewise; but I did not give them time to protrude out of the pelvis. I was called to attend the case at 8 o'clock A.M. She had been taken in labor about 12 o'clock in the morning, the first symptom being a gush of the liquor amnii, and this continued dribbling away until some time after my arrival. Cross births are usually characterized by irregular and defective pains, and in this case it was so. When first called, the uterus was but little open, and it was all I could do to reach the presenting part. I diagnosed presentation of the elbow. I remained with her several hours, pains very light indeed, and everything given to her to increase the pains and cause the opening of the os uteri sickened her so much that I ceased giving anything, and waited the natural dilatation, holding myself in readiness to act when the proper time arrived. About 5 P.M., the pains grew a little harder and the os was sufficiently open to introduce two or three fingers; then, I could prosecute my discoveries farther, and found it as first described. I found the umbilical cord was pulseless and, of course, the infant dead. I tried to reach the foot that did not present, but found it impossible. I then released the foot from its engagement over the arch of the symphysis pubis, and, by making intermittent tractions with the hand hold of the foot, succeeded in making the head sweep around from the right ilium, then to the fundus, and down the sacrum, by turning the face in that position after I got hold of the second foot, and had delivered it as far as the umbilicus.

*Transverse presentations* do not seem to be any more frequent among the poor than among those more pleasantly situated in life, and in affluent circumstances, though the contrary view has been advocated by some obstetricians. I know of no symptoms that would lead us to suspect a transverse presentation before the commencement of labor, after this commences, then, upon examination, if we find the part protruding, like a small oval tumor in the shape of the fingers of a glove, we may suspect something wrong. Another prominent symptom is a cessation, or nearly so, of pains, after the discharge of the liquor amnii. Digital examination alone is the only symptom that positively tells us that we are having a preternatural presentation to contend with. Now, should we be ignorant or incompetent, not knowing what to do, one of three things must happen: either the womb, by its continued action, would become ruptured; or the woman would very soon sink from exhaustion; or, lastly, the child, if small and the pelvis of the woman sufficiently large, might be forced down in a doubled state, and the violence usually done in such a case would usually result in the death of the mother.

No one, probably, condemns *meddlesome midwifery* more than I do; yet without the proper knowledge to know how to act and when to act, in this branch of our science, is criminal, and he who sticks his shingle up, advertising himself as a physician, should not only be an M.D. from some reputable school, but should possess good *common sense*, with a good tact for practicing this branch of our profession, for such troubles may present themselves to us at any time.

I will notice, very briefly, a case or two of placenta prævia. Eight or ten years ago, a man consulted me in regard to his wife. Five or six months had elapsed since her conception, and she was occasionally having spells of flowing. I told him my opinion was that the placenta was presenting, and the flowing would continue off and on until her confinement, and I impressed upon him the importance of having a physician in attendance at the first symptom she had of labor; but instead of complying with my instructions on this point, when the labor commenced they sent for her mother-in-law, who was an ignorant *virago* midwife. She officiated until she was entirely satisfied that she could accomplish nothing, and the woman would die from hemorrhage if undelivered. I was sent for in great haste, found the placenta presenting, and the umbilical cord prolapsed and out of the vagina ten or twelve inches. The child's belly was presenting, the head in the right and the feet in the left hypogastric region. She was flowing quite profusely, and, as the placenta was nearly detached, I immediately delivered it. I then immediately introduced my hand, grasped the feet, and delivered her in ten minutes time. The hemorrhage was profuse, though not sufficiently so to do any serious harm to the system, and she made a happy recovery. The child, of course, was dead, as I found the cord pulseless when I first entered the room.

Another case of placenta prævia came under my care in January, 1866. I was also consulted in this case, three or four months previous to her *accouchement*, in consequence of hemorrhage occurring from time to time. I diagnosed the case correctly, and told them frankly what they might expect, and enjoined them carefully to send for a physician at the first symptom of labor. They complied with my instructions, and I was sent for in good time. Every pain brought a gush of blood. I waited until the os uteri was dilated the size of a dollar. The parts were soft and quite dilatable; the placenta was immediately over the os. I administered chloroform, then partly ruptured the placenta and partly pushed it aside, introduced my hand in the womb, grasped the feet, and delivered my patient in fifteen or twenty minutes time of a dead infant. Not an unfavorable symptom presented after her *accouchement*, and on the first day of this month I delivered her of a fine, healthy boy, which came all right.

It is a very easy matter to turn and deliver, if we can select the right time and the right patient; but we rarely have this choice. As one author states, it is as easy to turn a child in utero, as it is a fish in a pail of water; but, in order to do it, we must be master of our business, and have the right time and the right kind of a pelvis. Select the time, if possible, before there is serious hemorrhage; do not wait too long for dilatation of the os, for if you do, your patient will sink from the loss of blood. If we can operate before the membranes are ruptured, we will find very little difficulty; the arm will prevent the liquor amnii from passing off, and the hand in the womb will find the infant floating in the water, which can be turned with ease and facility.

It is very rare, indeed, that both mother and child are saved in cases of placenta prævia; I have never had the good fortune to save both, neither have I ever had the misfortune to lose the mother, an occurrence which frequently happens to those poorly skilled in obstetrics, and occasionally to those who claim to be adepts in our profession.

Abortions occur so often, and are so frequently perplexing to the physician, that I would not do justice, even in this short report, were I not to treat of them in brief. The division of abortion, as divided by authors, into two stages, calling it abortion if it occurs before the sixth month, and premature labor if after that time, is, in my opinion, superfluous, and should be disregarded.

By criminal abortion, I mean where any drastic medicine is given to produce it, or where any instrument is introduced in os tincæ, breaking down the membranes, and soon bringing on abortion or premature labor. This is certainly a crime of great magnitude, when not produced to save the life of a woman. We are not authorized to operate on any case, without it is known that the woman has a deformed or contracted pelvis, and cannot be delivered without the assistance of instruments, and the child delivered dead. Every woman aborting, either naturally or from instrumental aid, runs a heavy risk of losing her life; and yet, physicians, knowing this great danger, will, for a few *paltry dollars*, yield to the solicitations of the gentler sex, or be urged on by the seducer, to perform this unnatural and infernal crime; a crime not second to the greatest in the catalogue of medical jurisprudence, but equal with murder in its first degree. In a word, we, as members of a great and good profession, should openly proclaim to the public that we denounce this great evil, and plainly let those persons know, who seek our aid in such cases, that neither money, love, high and aristocratic standing in society, or anything else can bribe us to commit this crime.

It matters but little, whether a woman aborts from natural or unnatural causes, the danger is about equally great, without the abortionist has given some severe drastic emenagogue or cathartic, thereby setting up inflammatory action in the womb and bowels, producing metro-peritonitis; or has used some sharp, penetrating instrument, and done violence to the os uteri, thence extending into the womb, and setting up the same active symptoms. A physician who is a gentleman, a moralist, or a Christian, will not permit such a stain to tarnish his character, whether known to the public, or alone to himself and his victim.

Our treatment of abortion, in ordinary cases, is well known to you all. If there is little or no flowing, keep your patient quietly in bed, and administer a mild opiate; when the pains have ceased, give some mild laxative, such as ol. ricini, sul. mag., or citrate of mag. But if we are not so successful as to check the premature action of the womb, then it is our services may become urgently demanded; we may have to use all our skill to check inordinate flowing, subue inflammatory action, etc. The lives of all women are very much jeopardized in cases of abortions; oftentimes the placenta is left undelivered for days, and sometimes for weeks, causing spells of hemorrhage, reducing the system from day to day, until anæmia and death is the result. One case, in particular, I will call your attention to. It occurred in my practice in the fall of 1865. The lady aborted six weeks before the placenta was removed. No physician was with her at the time and she thought the afterbirth came at the same time the fœtus did, but time proved the contrary. Hemorrhage continued from time to time, until it was delivered. I removed it with a pair of placenta forceps. My patient was perfectly anæmic, with a pulse of 140 to 150, and nearly unconscious at the the time I operated; but when I removed it, the bleeding ceased, and, under large doses of carb. ammo., quinine, and brandy, she rallied, and is now in good health.

I was sent for to see a patient in Stark County, in the winter of 1863, who was, or rather had been, under the care of an eclectic physician. Her husband informed me that she had miscarried, and he said the doctor told him the "afterbirth had come away all right." When I saw her, she seemed weak, pale, and excited, and had a pulse of 140; bowels tender and a little tympanitic. I made a digital examination, and found the placenta undelivered. I rendered the necessary aid, and soon my patient was convalescing. *What do you think of a physician who tells his patient she is delivered, and leaves her in this situation to die?*

One or two more cases of abortion, and I am through. A few years ago, I was sent for, in consultation, to see a case in Knox County. When I examined my patient, the attending physician told me that she had aborted at two months. On applying my hand over the pubic and hypogastric regions, I found the uterine tumor as large as after an ordinary labor. Why this unusual enlargement after an abortion? In due time the womb assumed its natural size and the woman promptly recovered.

Another very similar case I recently saw with Dr. SMILEY, of this place. A very near friend of his was taken with menorrhagia, which continued quite profuse from time to time, until the woman became anæmic and very prostrate. This condition lasted some two or three weeks. She was wakeful and irritable, and the doctor very properly became alarmed at her situation and requested me to call and see her. I did so, and on applying my hand to the bowels I found the uterine tumor filling the left hypogastric region full. The doctor thought she had not aborted at all, but considered it an unusual case of menorrhagia. If she had aborted, it could not have been more than from four to six weeks, as she had missed her menstrual flow but once. My first thought was that she had an ovarian tumor, and I interrogated the doctor closely in regard to its duration; he told me it could not possibly have existed more than two weeks. I then made a digital examination, placed my finger at the os tincæ as a guide, and introduced a bougie, fully four inches. In a day or two, she passed some membranous flakes, proving that she had aborted, as these were the membranes from around the fœtus. Strict quietude, recumbent position, warm fomentations, detergent washes, mild opiates, and tonics, were the means used for her restoration, and she is now able to sit up some, and the uterine tumor is rapidly diminishing. I consider these two cases as anomalous, and worthy a place in any report.

I would do injustice to myself and this Society, were I not to touch upon *puerperal peritonitis*, especially if I am able to report anything favorable in its treatment. Puerperal fever, or more properly styled puerperal peritonitis, is the great terror of the lying-in room. It is the disease at all times to be dreaded after ordinary, tedious, or instrumental labors. No physician performs his duty, in the lying-in chamber, after the labor is over, until he gives the patient and nurse strict and explicit orders in regard to her care, diet, etc. A little carelessness in diet, or the admission of drafts of cold air, or premature sitting up after delivery, may, at any time, develop this disease; especially will it do so when the malady is prevailing as an epidemic.

The disease, or rather its treatment, has become the opprobrium of medicine, and that, too, very properly. And why? Because, I think I can safely say, that nineteen patients out of every twenty, affected with *genuine* puerperal peritonitis, die in from two or three days to a fortnight after the attack. The old treatment of ARMSTRONG, so highly insisted on by him, *viz.*: free and repeated venesection, does not succeed in my hands, and I think any of you who have had patients with this disease, and have tried the heroic plan of ARMSTRONG, have come to the same conclusion that I have. This disease is so very fatal, that I think but very few patients recover under any treatment. I am of opinion that many cases reported as cured, especially in hospitals, are not genuine puerperal peritonitis, but merely metritis, connected with a high grade of fever. When this disease is once well developed, as known by a pulse running up from 120 to 160; frequent vomitings of a greenish fluid; retention of urine; extreme tenderness over the uterus, extending over the bowels; swelled and tympanitic state of the abdomen; lochia and milk checked or entirely suppressed, then it is we know a case of puerperal peritonitis. In its treatment, I would place calomel and the other mercurials along with blood-letting, *viz.*:—lay them aside for the treatment of some other diseases, for they are certainly not demanded in this disease.

I have the pleasure and gratification to communicate to you my treatment in two cases of well-marked puerperal peritonitis, during the present winter, both of whom recovered—one after a miscarriage of six months, the placenta being retained; and the other a natural primapara. Both cases had all the urgent symptoms I have mentioned, and my prognosis was, of course, unfavorable. My treatment was simple and, consequently, can be briefly stated:—It consisted in giving full portions of sul. morphine every three hours, to quiet the system and also the pain, and Norwood's tinct. ver. viride in drops, four to seven every three hours, to quiet the excessive action of the heart; thus alternating the two remedies every hour and a-half. When the stomach was much disturbed, I gave aqua cinnamomum or the essence of cinnamon in water. To quench the thirst, gave ice in small pellicles, orange, and lemonade, etc. In the active stage, I moved the bowels every three or four days by enema; I disapprove of drastics any time in the disease; and would only give ol. ricini and ol. terebinth. in the declining stage, and then when only imperatively demanded. Warm fomentations of hops should be constantly applied during the more active stage, to be succeeded in the stage of decline by a large epispastic, covering the whole abdomen. As soon as the stomach would retain nourishment, I gave milk, wine-whey, wine, etc., and at a still later period, I gave sugar-coated quinine pills as a tonic, every three or four hours. In the *main*, the above is my treatment, with very little variation, condensed in as few words as I am able to.

All of which is respectfully submitted.

ARTICLE XXX.

